# Meniscus Tissue Engineering Scaffolds: Biomaterials, Biofabrication, and Translation

**DOI:** 10.3390/polym18141717

**Published:** 2026-07-13

**Authors:** Wenbo Jin, Wenyu Ning, Ruoyu Wang, Danyang Zhao, Liangkun Lu, Fei Duan, Jian Yang, Cheng Zhang, Kedong Song

**Affiliations:** 1 State Key Laboratory of High-Performance Precision Manufacturing, Dalian University of Technology, Dalian 116024, China; jinwenbo19940820@163.com (W.J.); nwy20010304@mail.dlut.edu.cn (W.N.); ruoyu@mail.dlut.edu.cn (R.W.); zhaody@dlut.edu.cn (D.Z.); chengz9106@163.com (C.Z.); kedongsong@dlut.edu.cn (K.S.); 2 School of Mechanical and Electrical Engineering, Zhengzhou University of Light Industry, Zhengzhou 454002, China; 3 Ningbo Institute of Dalian University of Technology, Ningbo 315032, China; 4 The Affiliated Central Hospital, Dalian University of Technology, Dalian 116003, China; dlzxyyyj@163.com

**Keywords:** meniscus scaffold, biomaterials, biofabrication, 3D printing, clinical translation

## Abstract

The meniscus is a fibrocartilaginous tissue essential for load transmission, shock absorption, joint stability, and cartilage protection in the knee. However, its intrinsic healing capacity is severely limited, particularly in the avascular region and in complex defects, often resulting in persistent symptoms, functional impairment, and progressive joint degeneration. Although current clinical interventions, including meniscal repair, partial meniscectomy, allograft transplantation, and scaffold-assisted meniscal substitution, can provide symptomatic and functional improvement in selected patients, durable structural and functional restoration remains difficult to achieve. Meniscus tissue engineering has therefore emerged as a potential strategy for tissue preservation and functional reconstruction. This review synthesizes recent advances in meniscus tissue-engineered scaffolds, focusing on biomaterial systems, biofabrication strategies, and translational progress. Natural polymers, decellularized extracellular matrix, synthetic polymers, and composite materials are discussed according to their respective roles in biological regulation, mechanical support, structural organization, and clinical feasibility. Emerging biofabrication strategies are further analyzed with respect to geometric reconstruction, zonal organization, fibrous anisotropy, and their implications for scaffold evaluation. Finally, current in vitro, preclinical, and clinical evidence is critically examined to identify the key barriers that still limit long-term regeneration and clinical translation.

## 1. Introduction

The meniscus is essential for load transmission in the knee, shock absorption, joint stability, lubrication, and cartilage protection [1,2,3]. Structurally, the native meniscus is heterogeneous, combining fibrous features in the peripheral region with cartilage-like characteristics in the inner region. Its highly aligned collagen network sustains joint loading through a circumferential hoop stress-transmission mechanism [4,5,6]. Meniscal tears or tissue loss reduce the tibiofemoral contact area, increase local contact stress, and elevate the risk of secondary cartilage degeneration [7]. Epidemiological studies further indicate that meniscal injury is common in sports trauma, degenerative knee disorders, and postoperative degenerative progression, and contributes substantially to persistent symptoms and osteoarthritis development [8]. Healing is particularly limited in avascular, complex, and root lesions [9,10]. Current treatment paradigms have therefore increasingly emphasized tissue preservation and functional reconstruction [11].

Current clinical strategies include meniscal repair, partial meniscectomy, and meniscal allograft transplantation (MAT) [12,13]. Meniscal repair is generally preferred because it preserves native tissue, but its success depends strongly on lesion location, tear pattern, tissue quality, patient age, and concomitant joint pathology. Partial meniscectomy can rapidly relieve mechanical symptoms, but often at the cost of functional meniscal tissue and with increased long-term degenerative risk. For substantial meniscal deficiency or irreparable injury, MAT provides an replacement option, although outcomes remain influenced by donor availability, graft sizing, fixation, graft integration, and associated cartilage damage. More recently, collagen meniscus implants (CMI), polyurethane scaffolds such as Actifit, and other off-the-shelf implants have expanded the clinical options for partial meniscal substitution. However, symptomatic improvement does not equal durable structural or functional restoration, and evidence for long-term joint protection remains limited [14].

**Figure 1:**
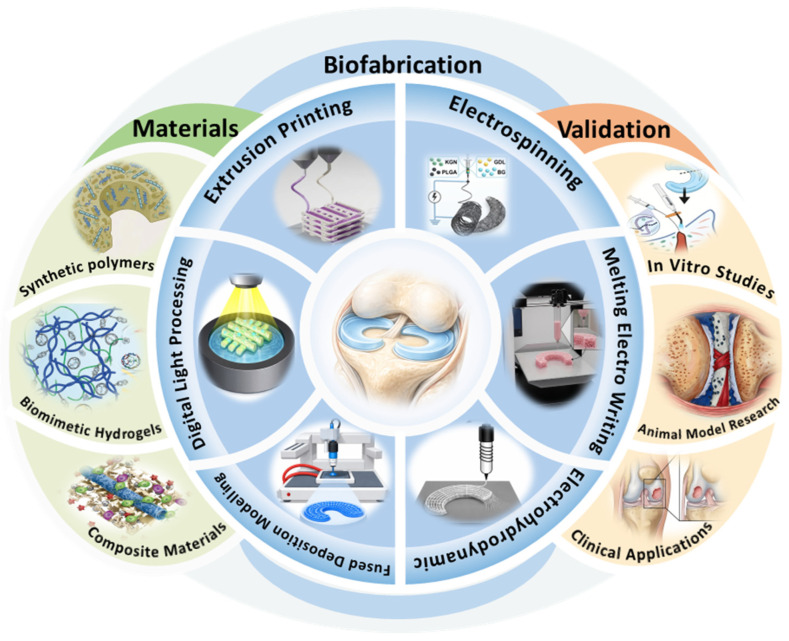
Conceptual framework of meniscus tissue engineering, highlighting the interplay among material systems, fabrication strategies, and multilevel validation and translational application.

Meniscus tissue engineering aims to move beyond defect filling toward multiscale structural and functional regeneration [15]. Recent advances in human pluripotent stem cell (hPSC)-derived organoids demonstrate how complex tissue architecture and function can be recapitulated in vitro, enabling disease modeling and drug screening that more closely reflect human physiology [16]. Such organoid-based strategies illustrate the importance of spatial organization and functional maturation, although the complexity of the native meniscus remains difficult to recapitulate [17]. Further progress in this field requires coordinated optimization of material systems, structural design, manufacturing routes, and biological responses [18]. Recent studies and reviews indicate that meniscus tissue engineering has moved beyond basic biocompatibility toward integrated requirements involving structural biomimicry, mechanical adaptation, tissue induction, and translational feasibility [19,20]. Although this programmable-scaffold study was not meniscus-specific and did not directly address meniscal anisotropy or joint loading, it supports the transferable idea that scaffold architecture can be quantitatively programmed before fabrication. In meniscus engineering, this principle should be extended beyond pore control toward geometry matching, load transfer, fiber orientation, and tissue ingrowth. Current research has expanded to dECM-based scaffolds, bioactive delivery systems, zonal reconstruction, and multimaterial design; however, long-term functional repair remains constrained by insufficient structural stability, incomplete host integration, and limited joint adaptation. Meanwhile, manufacturing has become a key determinant of scaffold architecture and structure–function performance in meniscus tissue engineering [21]. Building on these advances, this review does not aim to provide a purely material- or technique-centered survey. Instead, it organizes meniscus tissue-engineered scaffolds within a structure–function–translation framework that links ideal material requirements, biomaterial systems, biofabrication strategies, meniscus-specific structural reconstruction, multilevel validation, and translational barriers. This structure–function–translation perspective enables scaffold systems to be assessed in terms of their contributions to load-bearing support, zonal organization, fibrous anisotropy, host integration, and clinical feasibility. As illustrated in Figure 1, meniscus tissue engineering integrates biomaterials, fabrication, and multilevel evaluation to achieve functional reconstruction and joint protection.

## 2. Biomaterials for Meniscus Engineering

In meniscus tissue engineering, ideal scaffold materials should be evaluated based on the native structure–function relationship rather than any single parameter. An effective material system should provide adequate mechanical support for joint loading, enable anisotropic and zonal structural organization, promote cell adhesion and matrix formation, exhibit degradation kinetics synchronized with tissue remodeling, and remain compatible with reproducible fabrication and clinical translation. These criteria provide the basis for evaluating the biomaterial systems discussed in this section [22,23].

### 2.1. Natural Polymers and Biomimetic Hydrogels

**Figure 2:**
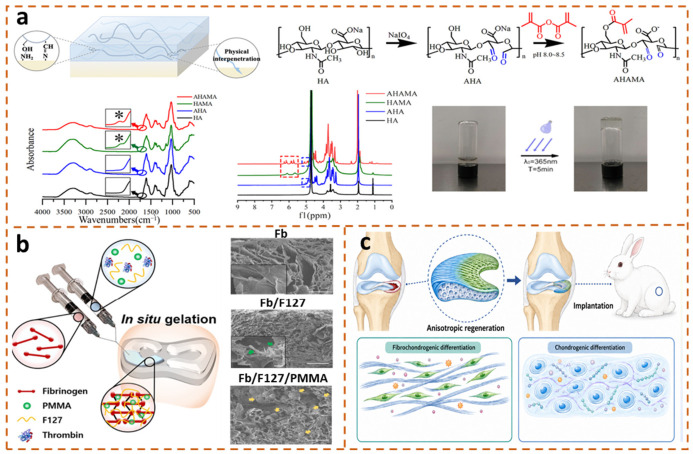
Representative hydrogel-based biomaterial systems for meniscus tissue engineering. (**a**) AHAMA hydrogel synthesis, characterization, and gelation. Reproduced from [29]. (**b**) Injectable Fb/F127/PMMA hydrogel with in situ gelation behavior. Reproduced from [37] (**c**) Zonal dECM-based biofabrication strategy for anisotropic meniscal regeneration.

Natural polymers and biomimetic hydrogels generally support cell adhesion and matrix formation, but they often lack sufficient mechanical stability for load-bearing meniscal reconstruction [24,25]. Collagen often requires reinforcement because of its limited structural stability, whereas GelMA provides relatively favorable printability and has been reported to maintain approximately 80% cell viability in comparative bioink studies under specific printing conditions. Polysaccharide hydrogels, especially HA and alginate, have also been widely investigated, with HA being widely used because of its roles in lubrication and microenvironmental regulation [26,27,28]. Chen et al. introduced aldehyde and methacrylate groups into HA to generate a derivative hydrogel precursor with both interfacial reactivity and photocuring capability, as verified by FTIR, 1H NMR, and photoinduced gelation assays (Figure 2a) [29]. Li et al. further emphasized that HA, collagen, alginate, and fibrin should be assessed within a scenario-based framework defined by defect type, load-bearing demand, and fabrication method [30], while Otsuki et al. showed that HA-related materials are increasingly being tailored to specific meniscal repair challenges such as irreparable tears [31]. Studies on alginate-based bioinks have demonstrated tunable gelation, cytocompatibility, and adjustable rheological properties, while meniscus-oriented bioink studies further indicate that alginate-based systems are more valuable as printable soft phases and cell carriers than as intrinsically meniscus-specific regenerative matrices [32,33,34].

Fibrin is suitable for in situ meniscus repair because of its injectability and adhesiveness, but its mechanical strength and structural stability remain limited [35]. Kim et al. developed an injectable fibrin/PEO semi-interpenetrating network hydrogel that improved tissue quality and mechanical recovery in a rabbit meniscus defect model [36], and An et al. further reinforced fibrin with Pluronic/PMMA, confirming that engineered reinforcement can improve the stability of injectable repair systems (Figure 2b) [37]. Silk fibroin-based systems offer mechanical robustness and, particularly when combined with collagen or PCL, show promise for partial meniscal replacement and improved construct integration [38,39]. By contrast, self-assembling peptide hydrogels remain limited in large-scale load-bearing applications, underscoring that effective meniscus engineering requires not only biomimetic composition but also organized collagen architecture and zonal structure [40,41]. Overall, natural polymers and biomimetic hydrogels are more valuable as bioactive modulatory components than as stand-alone load-bearing constructs. HA is closely associated with lubrication, matrix regulation, and cartilage-protective functions, whereas alginate is mainly useful as a printable carrier or rheology-adjusting phase. Fibrin-based systems are more suitable for localized defect filling, tear sealing, and minimally invasive repair than for full meniscal replacement. Silk fibroin provides comparatively better mechanical robustness, but it still usually requires structural design or combination with other materials to meet meniscal load-bearing demands. Therefore, the clinical relevance of natural polymers depends less on their intrinsic bioactivity and more on whether they can be mechanically reinforced and spatially organized within stable scaffold designs.

### 2.2. dECM and Tissue-Derived Biomaterials

Decellularized extracellular matrix (dECM) and tissue-derived biomaterials more closely recapitulate the native meniscal microenvironment by retaining tissue-specific ECM-derived biochemical and structural cues [42]. A representative example was the acellular meniscal ECM scaffold reported by Li et al., who used meniscus-derived dECM to construct a whole-meniscus regenerative scaffold and demonstrated its reparative potential in an animal model, thereby demonstrating the value of tissue-specific ECM in meniscal regeneration (Figure 2c) [43]. At the same time, this study revealed a limitation that has been repeatedly confirmed in later work: dECM alone rarely satisfies the simultaneous demands for large-scale construct formation, long-term structural stability, and mechanical restoration. Accordingly, subsequent studies shifted from direct use of dECM toward strategies that exploit its biological value within composite systems. Collectively, these studies indicate that dECM performs best in meniscus repair when it is integrated with appropriate scaffold frameworks and fabrication strategies.

**Figure 3:**
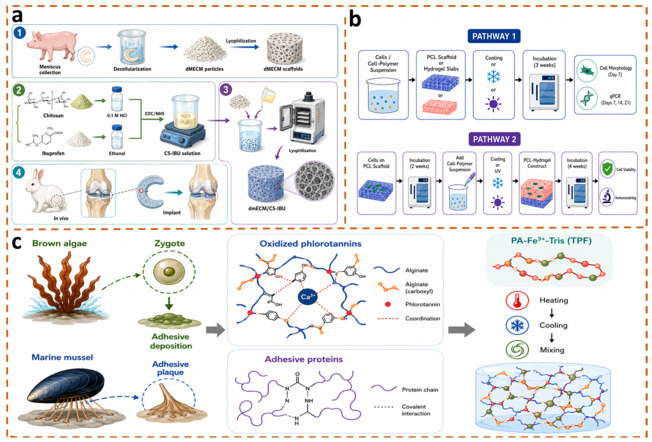
Representative dECM-based, composite, and bioinspired biomaterial systems for meniscus tissue engineering. (**a**) Preparation of a bioactive grafted dECM scaffold, including (1) meniscus collection, decellularization, dMECM particle preparation, and dMECM scaffold formation; (2) chitosan/ibuprofen modification; (3) fabrication of the dMECM/CS-IBU scaffold by lyophilization; and (4) in vivo implantation. (**b**) PCL-hydrogel composite construct for load-bearing support and zonal biological regulation. (**c**) Brown algal- and mussel-inspired adhesive/sealant design for interfacial fixation and tear repair.

The material form of dECM has expanded from sheet-like scaffolds to injectable systems and bioinks, with ECM hydrogels showing potential for stem cell delivery and meniscal repair. The role of dECM now extends beyond passive tissue mimicry to active regulation of the cellular microenvironment and signaling processes, and related ECM cues may also support cellular organization and fibrochondrogenic maturation [44,45]. Despite these advantages, dECM is accompanied by clear translational constraints. As emphasized by Crapo et al., Xing et al., and others, decellularization invariably requires balancing maximal removal of cellular components against preservation of critical ECM composition, architecture, and bioactive cues. Source-tissue variation, batch-to-batch inconsistency, residual DNA, sterilization methods, storage conditions, and subsequent blending processes can all affect final material performance and experimental reproducibility [46]. More importantly, dECM materials that perform well under laboratory conditions often face greater barriers to standardized preparation, large-scale production, and regulatory compliance. In this context, the inflammation-regulating elastic dECM scaffold reported by Ding et al. represents an important advance. By integrating elasticity and immunomodulatory design, this system enabled dECM to contribute simultaneously to structural support and microenvironmental regulation during regeneration (Figure 3a) [47]. These studies collectively suggest that dECM research is moving beyond the question of how much ECM can be retained toward how the regenerative behavior of dECM can be actively engineered. Compared with purified natural polymers, dECM provides more tissue-specific biochemical cues that support fibrochondrogenic differentiation, cell recruitment, and matrix remodeling. However, its biological relevance does not ensure sufficient mechanical strength or clinical reproducibility. Therefore, dECM should be regarded mainly as a bioactive component for meniscus repair, and its translational value depends on standardized processing, reliable preservation of ECM bioactivity, and integration with mechanically competent scaffold architectures.

### 2.3. Synthetic Polymers and Load-Bearing Scaffolds

Whereas natural hydrogels and dECM primarily provide a favorable biological microenvironment, meniscal reconstruction also requires materials capable of sustaining long-term load transfer and joint stabilization. Synthetic polymers therefore remain important in meniscus tissue engineering, particularly for patient-specific shape reconstruction, pore architecture design, shape retention, and mechanical support. Compared with natural materials, they generally offer superior processing stability, process controllability, and batch consistency, and are readily adapted to melt-based processing, fibrous fabrication, low-temperature deposition, and other three-dimensional manufacturing strategies. At the same time, their bioinertness, limited cell adhesion, and modest integration capacity have shifted their role from stand-alone substitutes toward composite scaffold platforms [48].

Among synthetic polymers, polycaprolactone (PCL) is widely used as a load-bearing material in meniscus research because its slow degradation, favorable melt processability, and stable shape retention are well suited for maintaining macroscopic geometry and long-term mechanical support. Subsequent work further demonstrated that printed PCL frameworks incorporating interlayer offsets and circumferential fiber bundles could regulate compressive and tensile response, while dual-hydrogel infiltration enabled biochemical compartmentalization between the peripheral and inner regions (Figure 3b) [49]. Together with related studies integrating PCL with hydrogels and biomimetic structural design, these findings show that PCL functions primarily as a programmable structural platform rather than simply as a high-modulus material.

Synthetic polymers often serve as structural platforms for integrating bioactive phases into implantable constructs. Polyurethane (PU) and related elastomers are particularly relevant because they may more closely approximate the cushioning and viscoelastic requirements of the meniscus than PCL [50], although clinical studies indicate symptomatic benefit but limited evidence of durable tissue quality or long-term reliability [51,52]. Other degradable polyesters, including PLA, PLGA, and PGA/PLLA, also provide tunable platforms, but their in vivo performance remains dependent on compatibility with tissue remodeling and the joint environment [53,54]. Overall, synthetic polymers offer advantages in mechanical support, shape retention, processability, sterilization, storage, and batch reproducibility, making them suitable for scalable scaffold manufacturing. However, their bioinertness, limited cell adhesion, insufficient host integration, and potential degradation–remodeling mismatch remain major limitations. PCL is useful as a structural framework, while PU and related elastomers may more closely approximate the cushioning and viscoelastic requirements of the meniscus. Thus, the key challenge is to couple synthetic-polymer-based mechanical support with anisotropic mechanics, biological activity, and long-term tissue integration.

### 2.4. Composite and Functionalized Materials

Composite and functionalized materials have become a major direction in meniscus biomaterials research because single-material systems rarely satisfy the combined requirements of bioactivity, mechanical support, manufacturability, degradation kinetics matched to tissue remodeling, and tissue integration. Rather than representing simple hard–soft combinations, these systems assign different functions to different material phases: thermoplastic frameworks provide shape retention and load-bearing support, hydrogel or dECM phases provide cell-compatible and tissue-specific biochemical cues, and incorporated bioactive agents regulate differentiation, inflammation, or matrix remodeling. This design logic is particularly relevant to the meniscus, where the scaffold must simultaneously maintain semilunar geometry, support circumferential–radial load transfer, promote regional matrix formation, and integrate with host tissue. Therefore, the value of composite systems should be assessed not by the number of incorporated components, but by whether each component contributes a defined mechanical, biological, architectural, or interfacial function [55]. Composite design in this context depends on coordinated control of composition, structure, and regenerative biology rather than simple hard–soft combinations.

Bioactive functionalization has also moved beyond simple factor incorporation toward control of release behavior, immune modulation, and host response. Li et al. incorporated kartogenin (KGN)-releasing microspheres into a PCL/meniscus-derived extracellular matrix (MECM) scaffold and showed that local delivery enhanced meniscal regeneration. In a related study, a sodium tanshinone IIA sulfonate (STS)-loaded PCL–MECM scaffold improved the regenerative microenvironment by regulating macrophage polarization [56]. In tear repair, sealing and restoration of local mechanical continuity may be more relevant than achieving a higher modulus alone. Pan et al. demonstrated this with a silk fibroin hydrogel adhesive for tear healing [57]. Liang et al. further developed an injectable self-healing hydrogel sealant inspired by mussel and brown algal adhesion, in which dynamic interfacial bonding enabled repositioning at room temperature and stronger adhesion at body temperature (Figure 3c) [58].

Interfacial engineering is critical in composite systems. Favorable intrinsic mechanics and bioactivity may not prevent failure under complex loading if stable transitions are lacking at the framework-soft phase interface, the host-scaffold interface, or between functional layers. The fibronectin-coated polyurethane scaffold studies by Arredondo and Torres-Claramunt showed that surface and interfacial bioactivity significantly affect cell adhesion and repair performance [59,60]. Interface design is therefore essential for durable functional performance. Composite and functionalized materials have also driven more integrated evaluation across materials, fabrication, and application. The reported implantable anisotropic 3D-bioprinted meniscus and the biomimetic scaffold with integrated structural components developed by Shen et al. show that materials research is moving beyond in vitro property comparison toward constructs that are manufacturable, implantable, and experimentally validated [61]. Li et al. similarly argued that further progress is more likely to arise from scenario-oriented synergy across material classes than from incremental improvement of single components [62]. Representative meniscal biomaterial systems are summarized in Table 1. Composite and functionalized materials are considered particularly relevant, as their translation is largely determined by fabrication strategies capable of controlling multiphase organization and multiscale structure. Based on this framework, current biomaterials for meniscus tissue engineering show complementary but incomplete profiles. Synthetic polymers provide superior structural support, processability, and batch reproducibility, but their intrinsic bioactivity and host integration remain insufficient. Composite and functionalized systems therefore represent a particularly promising direction because they allow different material phases to be assigned distinct mechanical, biological, architectural, and interfacial roles. However, they also introduce new challenges in fabrication reproducibility, mechanism attribution, quality control, sterilization, and regulatory evaluation. Future material design should therefore shift from searching for a universal material toward constructing function-integrated material systems tailored to specific meniscal repair scenarios.

## 3. Biofabrication of Meniscal Scaffolds

Fabrication strategies play a central role in translating biomaterial properties into meniscus-specific scaffold architectures and functional performance. Different material classes are commonly paired with different processing routes: soft and hydrated matrices are often processed through injectable systems, extrusion-based printing, embedded printing, or photocuring to maintain biological activity and cytocompatibility, whereas thermoplastic polymers are more suitable for molding, melt-based deposition, FDM, or MEW to provide structural support and shape stability. In more complex systems, fabrication serves as a means to integrate multiple functional components, enabling coordinated mechanical, biological, and structural features. Accordingly, fabrication strategies should be evaluated according to how effectively they translate material characteristics into desired structural and functional outcomes.

### 3.1. Traditional Scaffold Fabrication Methods

Before the widespread use of additive manufacturing, meniscal scaffolds were commonly fabricated using conventional porous-scaffold techniques, including freeze-drying, solvent casting/particulate leaching, salt leaching, gas foaming, phase separation, and molding-assisted approaches. These methods provided early platforms for meniscus reconstruction because they enabled the formation of highly porous structures that could support cell infiltration, nutrient transport, and extracellular matrix deposition. For example, porous polyurethane scaffolds prepared by freeze-drying combined with salt leaching showed tunable porosity, pore size, and interconnectivity, demonstrating the feasibility of polymeric porous scaffolds for meniscus replacement. Multilayered silk fibroin scaffolds and decellularized matrix-based porous scaffolds further indicated that traditional fabrication strategies could be adapted to generate meniscus-shaped or biologically active constructs with improved cytocompatibility and matrix-forming capacity [72,73].

However, these traditional methods also have clear limitations for meniscus-specific reconstruction. The resulting pore architecture is often governed by freezing, porogen distribution, gas nucleation, or phase separation processes, making it difficult to precisely control patient-specific geometry, circumferential–radial fiber organization, zonal gradients, and spatial distribution of cells or bioactive cues. In addition, many conventionally fabricated scaffolds show limited ability to reproduce the anisotropic load-bearing behavior of the native meniscus, especially hoop-stress transmission and regional mechanical heterogeneity. Therefore, although traditional scaffold fabrication methods established the foundation for porous meniscal scaffold development, their limited structural programmability motivated the transition toward 3D printing-based technologies, which allow anatomical reconstruction, programmable toolpaths, multimaterial organization, and more precisely controlled scaffold architectures.

### 3.2. Conventional 3D Printing

Conventional 3D printing established the basis for fabrication of patient-specific meniscal scaffolds through geometric reconstruction and programmable toolpath design. Several studies developed workflows from knee MRI segmentation to individualized printable meniscal models, demonstrating that scaffold design can be derived directly from patient-specific anatomical data rather than generic templates [74,75,76]. Filardo et al. extended this concept by producing a patient-specific meniscal prototype based on a human cell-laden scaffold, thereby linking medical imaging, digital modeling, and construct fabrication within a single workflow [77]. These studies advanced meniscal scaffold design beyond static shape replication by incorporating defect boundaries, thickness gradients, and local path planning into the fabrication process.

**Figure 4:**
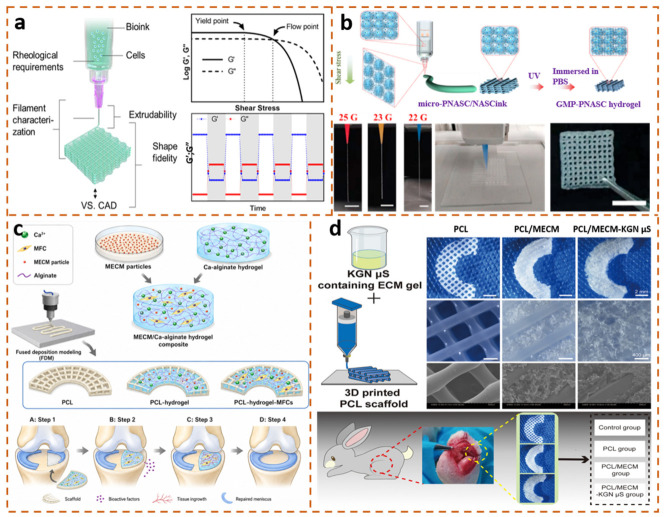
Representative fabrication strategies for meniscal scaffolds. (**a**) Rheological requirements and shape fidelity in extrusion-based printing. Reproduced from [79]. (**b**) Self-thickening hydrogel system for enhanced printability and structural support. Reproduced from [83]. (**c**) FDM-based fabrication of a PCL-hydrogel/MECM composite scaffold. Reproduced with permission from ACS [64]. (**d**) KGN-integrated PCL/MECM composite scaffold with structural characterization. Reproduced from [71].

Among conventional 3D-printing methods, direct ink writing (DIW) and related path-controlled extrusion systems are widely used for meniscal constructs. For curved and structurally organized scaffolds, post-deposition shape retention is often more critical than nominal resolution. Ribeiro et al. proposed a framework for shape fidelity, and Schwab et al. showed that printability depends on shear-thinning, yield stress, post-deposition recovery, and interlayer support [78,79] (Figure 4a). In meniscal scaffolds, these factors determine whether path-controlled printing generates organized architectures rather than simple layered deposition. Its main advantage lies in coupling external geometry with internal toolpath design [80]. However, DIW-based meniscal printing is still constrained by the trade-off between printability and mechanical competence: inks that print smoothly may lack post-print stability, whereas mechanically stronger formulations often reduce cytocompatibility or printing resolution.

Support-bath, freeform, and embedded-printing strategies further extend the design space for soft meniscal constructs. Highley et al. showed continuous deposition of low-modulus hydrogels in a self-healing support medium [81], while Hinton et al. addressed structural collapse through the FRESH strategy for complex soft-tissue geometries [82]. These approaches are particularly relevant for constructs in which high water content, cytocompatibility, and semilunar geometry must be preserved simultaneously. Xu et al. addressed this challenge with a self-thickening, high-modulus hydrogel printing system [83] (Figure 4b), which improved post-deposition support without markedly reducing printability and reproduced anisotropic mechanical characteristics. Compared with standard extrusion in air, embedded printing better preserves soft-material geometry, but it also introduces additional variables, including support-bath removal, interfacial contamination, diffusion-related boundary blurring, and scale-up complexity. Thus, conventional 3D printing should be regarded as a platform for geometric reconstruction rather than a complete solution for meniscal structure–function regeneration.

### 3.3. Extrusion-Based Biofabrication

Extrusion-based biofabrication is widely used in meniscus engineering because it enables cell encapsulation, multimaterial integration, and patient-specific shaping within a single fabrication workflow. Previous work highlighted persistent challenges in spatial complexity, mechanical matching, and human-scale fabrication, particularly when comparing dECM bioinks alone with co-extrusion using PCL frameworks [84]. In meniscal constructs, extrusion also introduces collapse, spreading, blurred interfaces, delamination, and shear-induced cell damage, which can compromise thickness gradients, pore architecture, and mechanical continuity. Extrusion-based fabrication should therefore be judged by structural fidelity and uniformity rather than printability alone. For meniscal applications, extrusion becomes clinically relevant when it can maintain construct-scale geometry, protect cell viability, and generate stable interfaces between mechanically and biologically distinct regions.

The transition from single-material extrusion to multimaterial deposition reflects a broader shift from shape validation toward integrated structure–function design. Jian et al. further demonstrated that combining material zoning with path design can improve cell distribution and fibrocartilage-like tissue formation while preserving overall structural integrity [67]. Chae et al. extended this concept by using a meniscus-derived bioink in 3D cell printing, showing that dECM-based bioinks become more effective when combined with polymeric support frameworks that preserve both biological signaling and structural integrity [85]. In meniscus engineering, multimaterial extrusion is most useful for spatially organizing distinct material functions within a single construct. Its advantage over single-material printing lies in functional partitioning: thermoplastic or reinforced phases provide shape and load support, whereas hydrogel or dECM phases provide biological regulation. However, this strategy may also introduce interfacial weakness, heterogeneous degradation, and more complex quality control.

In situ crosslinking is critical for extrusion-based meniscus fabrication because reliance on post-print bulk curing can compromise filament definition and interlayer stability. Here, shape fidelity should be treated as a process-dependent metric—including post-print recovery, wet-state retention, culture-stage contraction, and crosslinking-induced volume change—because geometric deviations directly affect stress distribution. Functional reconstruction therefore depends on coordinated control of material design, toolpath, crosslinking, and shape fidelity within a unified workflow [86]. Overall, extrusion-based biofabrication is one of the most versatile approaches for meniscal tissue engineering, but its translation will depend on whether multimaterial constructs can be produced reproducibly at clinically relevant size while maintaining cell viability, interfacial stability, and mechanical continuity.

### 3.4. Photocuring-Based Fabrication (SLA/DLP)

Compared with extrusion, photocuring techniques such as stereolithography (SLA) and digital light processing (DLP) provide higher resolution, sharper boundaries, and better reproducibility for fine soft-phase architectures. In meniscus engineering, the value of these techniques lies in reproducing complex geometry and rapidly stabilizing photoresponsive bioinks. The advantages of photocuring are therefore most evident in local structural refinement, high-resolution patterning, and rapid fixation of soft materials, rather than in the construction of an entire load-bearing meniscal substitute. In meniscus fabrication, photocuring cannot be directly extrapolated from cartilage printing or general hydrogel systems. Schuurman et al. and Levett et al. showed that, although photoresponsive networks improve early shape control, long-term stability still depends on crosslinking, hydration, and loading conditions [87,88]. Photocuring is therefore more suitable for soft-phase refinement and local stabilization than for stand-alone load-bearing reconstruction.

Accordingly, meniscus studies more often incorporate SLA/DLP-related photocuring into composite manufacturing workflows rather than treating it as an independent platform. The sequential gelation strategy proposed by Kesti et al. and the photo-crosslinking reinforcement of dECM bioinks reported by Jang et al. both follow this strategy: deposition is first completed within a printable rheological window, after which photochemical crosslinking rapidly establishes local structural stability [89,90]. Bandyopadhyay et al. showed that photocurable silk fibroin hydrogels are better suited for local defect filling, post-injection fixation, and patient-specific soft-phase reconstruction than for replacing load-bearing scaffold frameworks [91]. Thus, photocuring in meniscus fabrication mainly improves soft-phase precision, interfacial refinement, and construct stabilization, but remains limited by light penetration, material constraints, phototoxicity, and thickness-dependent curing. These curing-related limitations are particularly relevant for meniscus repair because the tissue has a large, curved, and mechanically demanding geometry. Therefore, general cartilage or hydrogel photocuring studies provide useful principles for shape fixation, cytocompatibility, and crosslinking control, but they cannot be directly treated as evidence for meniscal load-bearing reconstruction. In meniscus applications, photocuring should be evaluated according to whether it improves soft-phase stability, interfacial fixation, and regional matrix organization within a larger mechanically competent construct.

### 3.5. FDM-Based Composite Scaffolds

The principal role of fused deposition modeling (FDM) and related thermoplastic extrusion techniques in meniscus fabrication is to provide load-bearing scaffolds with stable geometry. Compared with direct printing of cell-laden hydrogels, FDM offers better path stability, dimensional control, and structural retention for thermoplastic polymers, particularly polycaprolactone (PCL) and related composites, making it well suited for forming semilunar contours, thickness gradients, and support networks. This FDM-based strategy typically separates mechanical support from bioactive function by introducing soft, tissue-specific phases after framework fabrication. A representative example is the zonal PCL/hydrogel scaffold reported by Bahcecioglu et al., in which PCL served as the geometric template and hydrogel phases provided region-specific biochemical cues. Li et al. further showed with a finely structured PCL/silk fibroin scaffold that an FDM framework provides not only structural support, but also a stable spatial platform for the subsequent incorporation of natural materials, cells, and functional cues [92]. Chen et al. developed a PCL–MECM hybrid scaffold, and Li et al. further introduced kartogenin (KGN)-releasing microspheres into a PCL–MECM construct, extending this strategy into an integrated platform that combines a structural framework, tissue-derived signaling, and controlled bioactive release [64,71] (Figure 4c,d). In this context, FDM serves as a structural basis for multifunctional composite systems rather than only as a method for fabricating mechanical scaffolds.

Another advantage of the FDM route is its strong compatibility with digital design and mechanical optimization. Li et al. showed using triply periodic minimal surface (TPMS)-based meniscal scaffolds that pore topology is not merely a geometric descriptor, but a determinant of stress distribution, load transfer, and subsequent cartilage protection [93]. Huebner et al. likewise demonstrated, in a more general fibrochondral context, that scaffold microstructure influences not only initial mechanics but also the mechanical properties and collagen organization of newly formed tissue in vivo [94]. These studies indicate that FDM-based scaffold design should be evaluated by load distribution, pore interconnectivity, tissue ingrowth potential, and cartilage-protective function, rather than by external shape accuracy alone. The limitations of FDM are also clear, as it is less suitable for reproducing fiber-scale organization, printing cell-laden soft materials, or forming compliant interfaces. Accordingly, FDM is now used mainly as a stable load-bearing platform integrated with hydrogels, bioprinting, photocuring, or fiber-directed fabrication. The clinical relevance of this approach is stronger than that of many purely soft hydrogel-printing approaches in terms of manufacturability, framework stability, and implant handling, but durable repair still requires improved host integration, anisotropic reinforcement, and mechanically reliable soft–hard interfaces.

### 3.6. Electric Field-Assisted Fabrication

**Figure 5:**
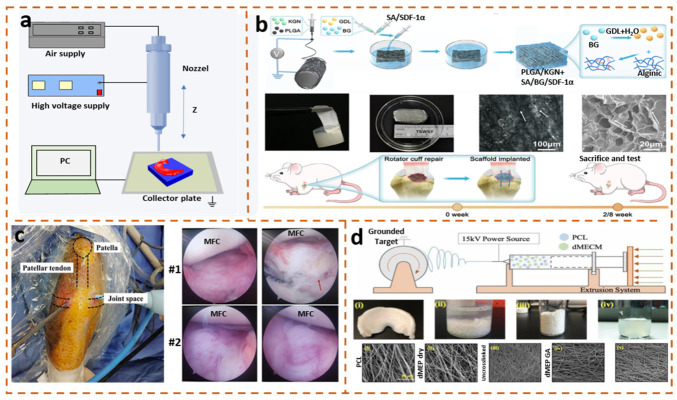
Electric field-assisted, nanofibrous, and bioactive scaffold strategies relevant to meniscus and fibrocartilage reconstruction. (**a**) Schematic illustration of electrohydrodynamic jet printing for meniscus scaffold fabrication. (**b**) Poly(lactic-co-glycolic acid) (PLGA)/kartogenin (KGN) electrospun membrane integrated with sodium alginate (SA)/bioactive glass (BG)/stromal cell-derived factor-1α (SDF-1α) hydrogel as a sequentially bioactive fiber–hydrogel composite system. The original study used this construct for fibrocartilage-interface-related rotator cuff tendon-to-bone repair, showing aligned electrospun fibers, porous hydrogel microstructure, stable membrane–hydrogel integration, and staged release of Si ions, SDF-1α, and KGN. Reproduced with permission from Wiley [99] (**c**) 3D-printed PCL meniscal scaffold combined with autologous synovium transplantation in a porcine subtotal meniscectomy model, showing the surgical procedure, scaffold fixation, synovium coverage, arthroscopic observation, regenerated meniscus-like tissue, and cartilage status during repair. Reproduced from [70]. (**d**) Decellularized meniscus extracellular matrix/PCL electrospun nanofibrous scaffold (dMEP), showing (**i**) gross scaffold morphology, (**ii**) dMECM gelation behavior, (**iii**) dMECM particle dispersion, (**iv**) uncrosslinked dMEP nanofibers, and (**v**) crosslinked dMEP nanofibers, together with enhanced meniscus cell spreading, proliferation, and fibrochondrogenic response compared with PCL-only scaffolds. Reproduced with permission from Elsevier [66].

The meniscus derives its load-bearing function from a hierarchical anisotropic architecture formed by circumferential primary fibers and radial tie fibers, making fiber organization a central target in meniscus engineering. Compared with extrusion-based biomanufacturing and FDM, which mainly address macroscopic shape and mesoscale architecture, electric field-assisted techniques are better suited for micro-/nanoscale fiber formation, alignment, and localized reinforcement. The main value of these techniques is therefore structural biomimicry at the fiber scale, rather than patient-specific whole-meniscus fabrication or direct clinical implant production. As schematically shown in Figure 5a, electrohydrodynamic (EHD) manufacturing uses an external electric field to induce jet formation, stretching, and deposition, providing a basis for directional micro-/nanofiber construction [95,96].

Electrospinning, as an early and widely studied representative, can generate ECM-like fibrous networks with tunable alignment. Studies by Ionescu and Mauck indicated that scaffold porosity, fiber organization, and pre-seeding affect maturation, cell infiltration, and integration [97], while later work from the Mauck group further demonstrated that nanofibrous architecture guides cell orientation and ECM organization through mechanical and adhesive cues [98]. These findings highlight that meniscus regeneration requires not only matrix deposition, but also restoration of collagen anisotropy. Compared with extrusion and FDM, electrospinning more effectively reproduces fibrous ECM-like microenvironments, but it is weaker in controlling construct thickness, patient-specific three-dimensional geometry, and uniform cell infiltration through dense fiber layers.

Subsequent studies increasingly focused on how fibrous architecture cooperates with bioactive cues to regulate tissue organization. Li et al. showed that the biological benefit of dECM incorporation in electrospun scaffolds depended on appropriate fiber configuration, which significantly affected cell behavior and scaffold bioactivity [65]. They further developed a composite system combining SDF-1α-loaded sodium alginate/bioactive glass hydrogel with KGN-loaded PLGA electrospun nanofibers. As shown in Figure 5b, this system effectively promoted fibrocartilage regeneration, collagen maturation, and mechanical recovery at the tendon-bone interface, providing a temporally programmed release strategy for tendon-bone repair [99]. Inspired by the developmental and regenerative processes of the meniscus, Yan et al. further established a fibrocartilage regeneration system that highlighted the importance of directional fibrous architecture in reproducing the developmental logic of native tissue, rather than merely reducing fiber diameter [70] (Figure 5c). Xia et al. prepared decellularized bovine meniscal matrix (dME) and combined it with electrospinning to fabricate dME/PCL composite nanofibrous scaffolds (dMEP). Comparative characterization and cell experiments against pure PCL scaffolds showed that these biomimetic nanofibrous scaffolds, produced using a green solvent-based system, simultaneously exhibited bioactivity and structural stability, as shown in Figure 5d [66]. Taken together, electrospinning is particularly valuable in meniscus engineering for constructing ECM-scale fibrous networks and anisotropic microenvironments, although its capacity for building large load-bearing structures remains limited. Therefore, electric field-assisted fabrication is best positioned as a strategy for fiber organization and microstructural reinforcement. The future role of EHD-based deposition will likely depend on integration with 3D printing, FDM frameworks, injectable hydrogels, or dECM-based systems, so that fiber-scale anisotropy can be linked to macroscale geometry, mechanical durability, and biological remodeling.

## 4. Evaluation and Therapeutic Progress

Evaluation in meniscus tissue engineering should be aligned with the underlying scaffold design rather than relying on uniform biological assays. Constructs emphasizing anatomical reconstruction are typically assessed by shape fidelity and dimensional stability, while load-bearing frameworks require attention to mechanical performance under relevant conditions. For zonal or multimaterial systems, region-specific matrix formation and interface integrity become key endpoints, and fiber-oriented designs are often examined for alignment and directional response. As scaffolds move toward translation, considerations such as integration, repair quality, and functional outcomes gain increasing relevance. In this context, evaluation criteria tend to reflect how material selection and fabrication approaches shape the resulting structure and its intended function.

### 4.1. In Vitro Evaluation

**Figure 6:**
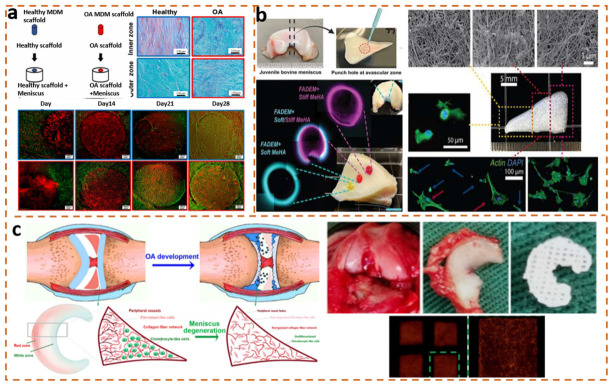
Representative in vitro and in vivo assessment of meniscus tissue-engineered constructs. (**a**) Healthy versus osteoarthritic meniscus-derived scaffolds in an in vitro defect model. Reproduced from [102]. (**b**) Stiffness-tunable FADEM hydrogels for zonal defects, with FE-SEM and cell morphology. Reproduced from [103]. (**c**) Phenotypic anisotropy during meniscal degeneration and evaluation of a printed biomimetic scaffold. Reproduced from [104].

In vitro studies in meniscus tissue engineering are commonly used to assess whether specific material–structure combinations support fibrochondrogenic maturation, rather than merely to confirm construct fabrication. Thus, evidence based solely on cell viability or short-term proliferation is insufficient; more informative evaluation should address cell morphology, phenotype maintenance, ECM deposition, tissue integration, construct contraction, and wet-state mechanical stability. A key issue is the compatibility between the cell source and scaffold microenvironment. Canciani et al. showed that the availability of human cell sources does not necessarily indicate their suitability for meniscus engineering, as different cells exhibited distinct proliferation, phenotype retention, and matrix-forming capacities in type I collagen gel scaffolds [100,101]. Firoozi et al. further demonstrated that, although both healthy and osteoarthritic meniscus-derived matrix scaffolds supported cell growth, healthy matrix promoted more robust explant integration [102] (Figure 6a). These findings indicate that in vitro assessment has shifted from simple cytocompatibility tests toward repair-relevant evaluation of matrix formation, integration, and functional maturation.

Studies addressing meniscal zonal heterogeneity have further refined this framework. Lee et al. developed tunable meniscus-derived ECM hydrogels and showed that both matrix origin and network parameters influenced cell behavior in a zone-dependent manner, with adult-derived dECM hydrogels more favorable for fibrochondrogenic phenotype induction and stiffness further modulating cell morphology and regional characteristics [103] (Figure 6b). Sun et al. further demonstrated that anisotropic, implantable bioprinted meniscal constructs influence subsequent phenotypic differentiation and ECM organization through zonal path design and material distribution, rather than through initial geometry alone [104] (Figure 6c). These studies show that structural biomimicry in vitro should be understood as a means of directing later tissue maturation rather than as simple geometric imitation. A further trend is the shift from static cytocompatibility assays to function-oriented evaluation. Live/dead staining, proliferation, and limited gene expression provide only preliminary evidence, whereas more informative studies assess collagen I/II balance, proteoglycan deposition, integration strength, construct shrinkage, wet-state mechanics, and zonal ECM distribution. Nevertheless, in vitro models remain useful for screening and mechanistic analysis, as they cannot fully reproduce physiological loading, synovial fluid, inflammation, or tissue-interface responses [105].

Static culture systems do not fully reproduce the multiaxial mechanical environment experienced by the native meniscus. Meniscal constructs are exposed in vivo to compression, circumferential tension, shear, hydrostatic pressure, and interstitial fluid flow. Accordingly, perfusion, cyclic compression, hydrostatic pressure, and combined compression–shear bioreactors have been explored as platforms for improving nutrient transport and promoting functional maturation before implantation. Controlled mechanical conditioning may enhance matrix synthesis, collagen organization, and construct mechanical properties; however, its effects depend strongly on cell source, scaffold composition, loading magnitude, frequency, duration, and the maturity of the construct at the time of stimulation. Excessive or prematurely applied loading may instead cause cell damage, matrix catabolism, interface delamination, or permanent deformation. Therefore, bioreactor evaluation should report not only nominal loading parameters, but also local strain distribution, cell viability, regional ECM formation, interfacial integrity, and mechanical retention after conditioning. At present, the lack of standardized loading protocols and anatomically relevant test systems limits direct comparison among studies and remains an important barrier to translation.

### 4.2. In Vivo Evaluation

**Figure 7:**
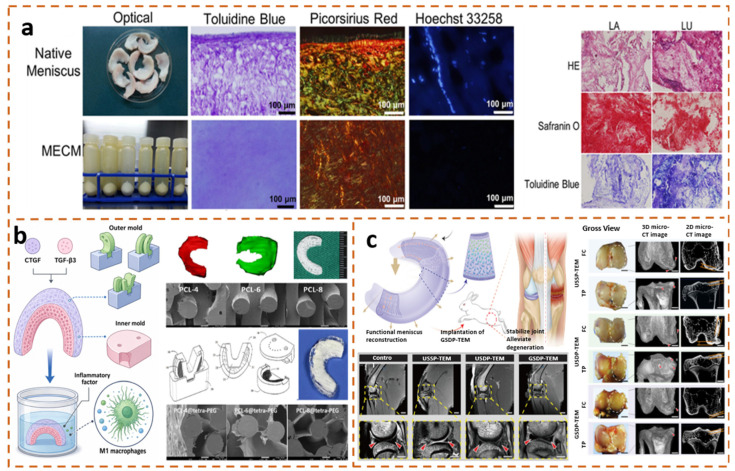
Preclinical in vivo evaluation of meniscus tissue-engineered constructs. (**a**) Native meniscus versus MECM and outcomes after 4 weeks of subcutaneous implantation. Reproduced from [108]. (**b**) Gradient-porosity PCL scaffold with heterogeneous architecture and cytocompatibility. Reproduced from [69]. (**c**) Biomimetic neo-meniscus reconstruction and post-implantation MRI evaluation. Reproduced from [113].

In vivo evaluation assesses engineered meniscal constructs under more realistic joint conditions, including load transmission, tissue integration, inflammatory response, scaffold degradation, and cartilage protection. However, its translational value depends strongly on model selection. Common rabbit meniscectomy or segmental defect models only partly reproduce human meniscal size, loading, and chronic pathology; therefore, animal evidence should be interpreted in relation to model type, defect complexity, animal age and health status, and follow-up duration. A major line of evidence comes from acellular or cell-free or acellular bioactive scaffolds for in situ regeneration. Lee et al. showed that a protein-releasing polymer scaffold induced endogenous fibrochondrogenic regeneration in a sheep model [106], while Gao et al. reported meniscus-like tissue formation and partial cartilage protection using an electrospun fiber-reinforced scaffold in rabbits [107]. Guo et al. further reported that an acellular 3D-printed PCL-MECM scaffold supported meniscus-like regeneration in rabbits and demonstrated structural reconstruction and cartilage-protective potential in sheep [108] (Figure 7a). Cell-assisted strategies have also shown promise: injectable fibrin hydrogels, ADSC sheets, and related systems improved neo-tissue formation, tensile strength, and load-distribution function in rabbit meniscal defect models [109,110]. Together, these studies suggest that effective meniscus repair requires not only histological regeneration, but also evidence of structural reconstruction, mechanical recovery, and joint-protective function.

More recent animal studies increasingly incorporate functionalization and immune regulation. Xu et al. developed a gradient porous anisotropic scaffold with spatiotemporally controlled protein release, anti-inflammatory regulation, and antioxidant activity, achieving tissue remodeling more similar to the native meniscus [69] (Figure 7b). Wang et al. used a thermosensitive HA/chitosan hydrogel loaded with TGF-β1 for full-thickness meniscal tear repair in rabbits and observed improved cell migration, ECM formation, and local healing [111]. Hao Wang et al. further developed a peptide-modified decellularized skin matrix scaffold that enhanced MSC recruitment, matrix formation, and cartilage protection in a rabbit meniscectomy model [112]. Du et al. designed a tissue-engineered meniscus with gradient rhombic pore architecture that promoted biological and biomechanical heterogeneity through topological self-induction and yielded favorable meniscal regeneration and cartilage protection in rabbits [113] (Figure 7c). Ma et al. further showed that immunomodulatory dECM scaffolds and PCL-fibrin composites can improve tissue reconstruction and reduce secondary cartilage degeneration [114]. Otsuki et al., using a miniature pig model to evaluate a PGA scaffold, further highlighted the importance of moving toward large-animal models that better approximate clinical loading conditions [115]. Animal evidence remains limited by species differences, simplified defect models, and short follow-up. Current studies support regenerative potential, but do not yet establish clinical equivalence or long-term joint protection.

Long-term in vivo evaluation should further consider remodeling as a dynamic balance among scaffold degradation, mechanical retention, neotissue formation, host integration, and cartilage protection. Early defect filling or positive histological staining does not necessarily indicate durable restoration of meniscal function, because the scaffold must maintain sufficient mechanical support while newly formed tissue gradually acquires load-bearing capacity. Premature degradation may lead to construct collapse, extrusion, fixation failure, or loss of stress-transfer function, whereas excessively slow degradation may limit tissue ingrowth, prolong foreign-body reactions, or maintain a mechanical mismatch with host tissue. Therefore, future in vivo studies should evaluate scaffold degradation and neotissue formation simultaneously, including scaffold-volume retention, extrusion, host integration, inflammatory response, regional collagen organization, mechanical testing of retrieved constructs, and secondary cartilage degeneration. Extended follow-up in large-animal models is needed to determine whether engineered constructs can achieve functional remodeling rather than short-term tissue filling alone.

### 4.3. Clinical Translation

Compared with in vitro and animal studies, clinical evidence for meniscus tissue engineering remains limited and is still dominated by off-the-shelf scaffolds and replacement implants rather than highly biomimetic, cell-laden, or multimaterial biofabricated constructs. Existing studies mainly involve collagen meniscus implants (CMI), polyurethane scaffolds such as Actifit, and synthetic replacement devices such as NUsurface, but heterogeneous outcome measures, follow-up durations, imaging criteria, patient selection, concomitant procedures, and limited randomized controls constrain direct comparison [116,117]. Therefore, translational potential should be evaluated not only by symptomatic improvement but also by structural durability, cartilage protection, failure mechanisms, regulatory feasibility, cost burden, and commercialization pathways. Recent systematic reviews indicate that these implants can improve symptoms and function in selected patients, but available evidence has not yet established durable cartilage protection, clear failure-mode differences, or robust superiority among implant types [118,119].

CMI is among the earliest clinically investigated scaffold strategies. Rodkey et al. showed that collagen meniscus implants supported tissue ingrowth and improved selected clinical outcomes compared with partial meniscectomy [120]. However, later long-term studies reported less consistent results, showing acceptable function in selected patients but no consistent superiority over meniscectomy or stable chondroprotection [121].

Similarly, polyurethane scaffolds such as Actifit have shown pain reduction and functional improvement over 5–10 years, but with clinically relevant failure rates, abnormal MRI appearance, inconsistent cartilage protection, and progressive degeneration in the treated compartment [122,123]. These findings suggest that CMI and PU scaffolds are better viewed as symptom-relieving and function-improving options for selected indications than as reliable substitutes for the native meniscus.

Synthetic implants such as NUsurface expand the clinical discussion from partial scaffold replacement to meniscal substitution. McKeon et al. reported early improvements in pain and functional scores compared with non-surgical treatment [124], and Zaslav et al. showed greater improvement at the 1-year follow-up in KOOS pain, function, and quality-of-life subscales after medial meniscus replacement [125]. Nevertheless, Reale et al. found no clear overall difference between CMI and Actifit [126], while Ghisa and Zaslav emphasized that current replacements differ substantially in indications, fixation strategies, and complication profiles [127]. Jahani et al. and Bian et al. further indicated that comparative evidence remains limited and that no implant has yet shown definitive long-term advantage [128].

Clinical exploration of cell-based or cell-augmented strategies remains at a more preliminary stage. Whitehouse et al. reported the early clinical use of autologous MSCs with a collagen scaffold for avascular meniscal tear repair, suggesting initial safety and local repair feasibility [129]. Vangsness et al. observed increased meniscal volume and pain improvement after allogeneic MSC injection following partial medial meniscectomy [130]. However, small cohorts, variable pathology, incomplete mechanistic evidence, and confounding from concomitant procedures, inflammation, or spontaneous recovery limit their strength of evidence [131]. Current clinical evidence supports short- to mid-term symptomatic and functional benefits, but long-term durability, stable chondroprotection, and restoration of native meniscal function remain unproven, underscoring the need for more rigorous translation-oriented evaluation frameworks.

## 5. Challenges and Outlook

Despite substantial progress in biomaterials, structural design, fabrication strategies, and preclinical validation, meniscus tissue engineering remains at a transitional stage between technical feasibility and clinical translation. Biomaterial selection constrains feasible fabrication routes, fabrication strategies translate material properties into scaffold architectures, and these architectures further determine mechanical stability, cell infiltration, matrix organization, tissue integration, and repair outcomes. Therefore, the central challenge is not any single material property or fabrication resolution alone, but whether material selection, manufacturing strategy, scaffold structure, and evaluation criteria can be coherently matched to reproduce the integrated structure–function behavior of the native meniscus.

The first challenge is to restore structure–function coupling across scales. Although semilunar geometry, zonal material distribution, and partial mechanical reinforcement have been increasingly reproduced, current constructs still insufficiently mimic the circumferential–radial fiber network, hierarchical transitions, horn-related mechanical continuity, and time-dependent response of the native meniscus. Unfavorable outcomes in some reinforced scaffold models and general fibrochondral studies further indicate that similarity in static modulus, cell viability, or matrix deposition does not necessarily translate into physiological load transfer or durable in vivo function. Thus, clinical repair or substitution potential requires coordinated control over macroscopic geometry, mesoscopic zonal organization, microscopic fiber alignment, and interfacial integration.

A second challenge is the lack of a coherent evidence chain matched to scaffold design. In vitro, animal, and clinical studies use different endpoints and evaluation windows, ranging from viability and ECM deposition to histological outcomes, mechanical testing, MRI findings, pain scores, and functional scores, which weakens comparability. Animal models also remain imperfect surrogates for clinical disease; Peng et al. noted the continued reliance on small-animal models, especially rabbits, despite major differences from humans in tissue scale, gait, loading, and chronic pathology [132]. Evaluation should therefore be linked to scaffold design intent: geometry-oriented scaffolds should be assessed for shape fidelity and dimensional stability; load-bearing frameworks for compressive, tensile, wet-state, and fatigue-related mechanical properties; zonal constructs for region-specific matrix formation; fiber-directed scaffolds for alignment and directional mechanics; and translation-oriented implants for host integration, cartilage protection, failure modes, and clinically meaningful outcomes.

A third challenge concerns manufacturing consistency and translational pathways. Laboratory constructs often depend on complex combinations of material sources, fabrication steps, crosslinking conditions, cell states, and multiparametric process control, complicating scale-up, standardization, sterilization, storage, reproducibility, and regulatory evaluation, especially for dECM-based, cell-based, bioactive-molecule-loaded, or multicomponent-based systems. Patient-specific scaffold design and defect-oriented anatomical reconstruction are becoming increasingly feasible with imaging-based modeling and additive manufacturing [133], and MSC-, HA-, or biologic-enhanced strategies may improve local repair, but their effects remain dependent on carrier design, delivery route, dose window, and defect context [134]. Future progress will therefore require a coherent framework that integrates cross-scale structural design, material–fabrication matching, clinically relevant evaluation models, manufacturing consistency, and patient-specific platforms that remain compatible with regulatory evaluation.

## 6. Conclusions

Meniscus tissue engineering has progressed from simple defect filling toward function-oriented scaffold reconstruction that requires coordinated optimization of biomaterials, fabrication strategies, scaffold architecture, biological response, and translational feasibility. This structure–function–translation synthesis summarizes recent advances in meniscus tissue-engineered scaffolds from a structure–function–translation perspective, emphasizing that effective meniscal repair cannot be achieved through material selection, fabrication precision, or biological stimulation alone. Instead, scaffold design should integrate mechanical support, zonal organization, fibrous anisotropy, tissue-specific bioactivity, and clinically relevant evaluation.

Current evidence indicates that natural polymers, dECM-based materials, synthetic polymers, and composite systems each provide distinct but incomplete contributions, while traditional fabrication methods, 3D printing, extrusion-based biofabrication, photocuring, FDM, MEW, electrospinning, and other emerging strategies provide different degrees of control over scaffold structure and function. Future progress will depend on rational material–fabrication matching, more predictive in vitro and in vivo evaluation systems, stronger preclinical–clinical evidence chains, and clearer regulatory and commercialization pathways. Therefore, the development of clinically translatable meniscal scaffolds should shift from isolated material or process optimization toward integrated design frameworks capable of supporting durable joint function and long-term cartilage protection.

## Figures and Tables

**Table 1:** Representative biomaterial systems for meniscus tissue engineering.

Category	Representative Materials	Crosslinking	Fabrication	Main Advantages	Key Limitations	Key Outcomes	Ref.
Natural polymers and biomimetic hydrogels	Collagen scaffolds	Chemical or physical crosslinking	Scaffold fabrication	Good cell adhesion; native ECM-like composition	Wet shrinkage; weak mechanics; limited durability	Supported cell adhesion; limited dimensional stability	[17,24,30]
	Bioinks based on agarose, alginate, GelMA, and PEGMA	Thermal, ionic, or photo-crosslinking	3D bioprinting	Printable hydrogel matrix; supports cell encapsulation	Mechanical strength and long-term stability remain limited	GelMA showed highest print fidelity; acceptable viability	[27]
	GelMA-type hydrogels	Photo-crosslinking	Bioprinting	Photocurable; good printability; tunable shape formation	Mechanical strength and long-term stability remain limited	Tunable shape formation; fibrocartilage-oriented design	[63]
	Modified HA precursor	Aldehyde and methacrylate modification; photocuring	In situ photocuring	Wet-environment curing; interfacial reactivity; lubrication-related bioactivity	Limited bulk mechanics; requires chemical modification	Rapid curing; improved interfacial reactivity	[29]
	HA-related meniscal systems	Derivatization or composite formulation	Injectable or scaffold-based systems	Lubrication support; microenvironment regulation; cartilage-protective potential	Scenario-dependent effect; weak independent structural support	Defect-dependent repair effects	[28,31]
	Alginate-based bioinks and composites	Ionic crosslinking with secondary reinforcement	Bioprinting	Excellent rheology control; rapid gelation; manufacturing compatibility	Limited intrinsic bioactivity; poor long-term stability	Printability; cell encapsulation compatibility	[33]
	Pluronic–PMMA reinforced fibrin	Dual-syringe gelation; semi-IPN; PMMA reinforcement	Injectable in situ repair	Injectable; adhesive; improved matrix stability	Limited durability under sustained mechanical loading	Improved mechanics and repair	[37]
	Silk fibroin scaffold	Porous scaffold formation	Scaffold implantation	Better mechanical robustness; good biocompatibility	Coating stability and mechanical matching remain concerns	Biocompatibility; mechanical robustness	[38]
	Collagen-coated sponge–silk scaffold	Freeze-drying; collagen coating	Scaffold fabrication	Improved surface bioactivity and tissue integration	Coating stability and mechanical matching remain concerns	Improved tissue integration	[39]
	Self-assembling peptide hydrogel	Peptide self-assembly	Injection or defect implantation	Injectable; biomimetic; supports matrix formation	Weak load-bearing capacity; limited large-scale application	Defect repair; collagen I/II matrix formation	[40]
Decellularized matrix and tissue-derived biomaterials	Acellular meniscal ECM scaffold	Decellularization and scaffold processing	Whole-meniscus scaffold fabrication	Tissue-specific ECM cues; native biochemical information	Weak mechanics; source variability; standardization difficulty	In vivo meniscal repair response	[43]
	PCL–MECM hybrid scaffold	Ionic gelation of MECM phase	PCL printing with hydrogel infusion	Combines load-bearing support with dECM bioactivity	Interfacial integration and fabrication complexity	Combined support and bioactivity	[64]
	dECM electrospun or nanofibrous systems	Fiber formation	Electrospinning	ECM cues with fibrous architecture	Limited 3D shape control and scalable fabrication	Improved cell response and tissue relevance	[65,66]
	ECM hydrogel for BMSC delivery	ECM digestion and thermal gelation	Injectable delivery	Injectable cell delivery; supports fibrochondrogenic repair	Poor mechanical retention; variable gelation behavior	Fibrochondrogenic repair; reduced joint degeneration	[44]
	Elastic immunomodulatory dECM scaffold	Freeze-drying; dehydrothermal treatment; EDC/NHS grafting	Porous scaffold implantation	Elastic support; immune regulation; tissue-specific signaling	Complex preparation; reproducibility and long-term validation needed	Inflammation regulation; meniscus-related phenotype expression	[47]
Synthetic polymers and load-bearing scaffolds	PCL scaffold platform and zonal PCL–hydrogel constructs	Melt deposition; agarose cooling; GelMA photocrosslinking	3D printing with zonal infiltration	Stable shape retention; zonal design; mechanical support	Bioinert framework; phase bonding and degradation mismatch	Mechanical support; regional matrix formation	[49,63]
	Biomimetic PCL-based constructs	Melt-based scaffold fabrication	3D bioprinting or low-temperature deposition	High designability; controllable architecture; good processability	Limited bioactivity; long-term integration remains uncertain	Improved construct architecture	[61,67]
	PU scaffold	Porous scaffold formation	Implantation and clinical follow-up	Viscoelastic cushioning; clinically relevant partial replacement	Unclear tissue regeneration; indication-dependent outcomes	Symptom improvement; MRI-based remodeling concerns	[50,51,68]
	PLDLA–PCL-TMC porous scaffold	Thermoplastic porous scaffold formation	Solvent casting and particulate leaching	Improved toughness; tunable pore structure	Limited biological cues; tissue ingrowth remains challenging	Improved scaffold processability and support	[52]
	Fiber-reinforced total meniscal scaffold	Fiber-reinforced scaffold formation	Total meniscal implantation	Large-scale reinforcement; improved structural support	Native anisotropy and biological integration remain incomplete	Tissue support; reinforcement feasibility	[53]
Composite and functionalized materials	Gradient porous scaffold with spatiotemporal release and immunoregulation	Tetra-PEG gelation; zonal factor loading	MRI-guided modeling and gradient printing	Zonal biomimicry; controlled release; immune modulation	Complex fabrication; release control and validation challenges	Heterogeneous biomimicry; improved microenvironment	[69]
	Regeneration-inspired material and structural system	Scaffold implantation with synovial coverage	3D-printed scaffold and fixation	In vivo maturation; structural and mechanical restoration	Surgical dependence; biological variability; reproducibility concerns	Improved mechanical restoration	[70]
	KGN microspheres in PCL–MECM scaffold	Microsphere loading and composite assembly	Composite scaffold fabrication	Local bioactive release; enhanced differentiation	Dose control, release safety, and long-term efficacy need validation	Enhanced differentiation and matrix deposition	[71]
	STS-loaded PCL–MECM scaffold	Drug loading and composite assembly	Composite scaffold fabrication	Immunoregenerative regulation; macrophage polarization	Immune response complexity; release kinetics require control	Macrophage polarization; microenvironment modulation	[56]
	Silk fibroin adhesive hydrogel	Visible-light curing; boronate, H-bonding, and β-sheet interactions	In situ sealing and curing	Wet adhesion; tear sealing; local continuity restoration	Long-term adhesion under cyclic loading remains uncertain	Improved adhesion; tear repair	[57]
	Injectable self-healing sealant	Dynamic bonding: H-bonding, Schiff or Michael addition, Fe^3+^ coordination	Injectable sealing	Injectable; self-healing; repeatable adhesion	Mechanical retention and in vivo durability remain unclear	Self-healing; injectability; repeatable adhesion; healing promotion	[58]

Abbreviations: BMSC, bone marrow-derived mesenchymal stem cell; ECM, extracellular matrix; GelMA, gelatin methacryloyl; HA, hyaluronic acid; MECM, meniscus-derived extracellular matrix; PCL, polycaprolactone; PU, polyurethane; STS, sodium tanshinone IIA sulfonate.

## Data Availability

No new data were created or analyzed in this study.
